# Building an interactive dashboard to visualize institutional open access publishing trends

**DOI:** 10.5195/jmla.2026.2340

**Published:** 2026-04-01

**Authors:** Emily F. Gorman, Nicole Shelawala, Amy Yarnell

**Affiliations:** 1 efgorman@hshsl.umaryland.edu, Research and Education Librarian, Health Sciences and Human Services Library, University of Maryland, Baltimore, MD; 2 nshelawala@hshsl.umaryland.edu, Research and Education Librarian, Health Sciences and Human Services Library, University of Maryland, Baltimore, MD; 3 ayarnell@hshsl.umaryland.edu, Head of Data and Bioinformation Services, Health Sciences and Human Services Library, University of Maryland, Baltimore, MD

**Keywords:** Open Access Publishing, Scholarly Communications, Data Visualization, Interactive Dashboards

## Abstract

As part of an effort to seek sustainable support models for Open Access (OA) publishing, the University of Maryland, Baltimore (UMB), Health Sciences and Human Services Library's (HSHSL's) Scholarly Communications Committee developed an interactive dashboard to visualize university-wide OA publishing trends. Using publication data exported from Scopus and visualized in Microsoft Power BI, the dashboard displays five years of publishing trends by OA model, publisher, journal, school, and citation count. The dashboard is fully interactive, allowing users to filter results based on school, OA model, and year.

The design of the dashboard was iterative, with planning discussions taking place in Summer 2024, data model development and initial data collection in Fall 2024, refining of the visualization and data model in early Spring 2025, and the publication of the final dashboard to our website in April 2025. The dashboard continues to be refined and improved based on feedback from stakeholders, and the project team plans to incorporate data on publishing costs in Spring 2026.

The project was designed for sustainability and adaptability, with a documented workflow that will be easy for future committees to implement. This innovative, replicable approach supports informed decision-making around OA publishing and provides a model that can be adopted by other academic health sciences libraries.

## BACKGROUND

Researchers at the University of Maryland, Baltimore (UMB) often express concerns about meeting Open Access (OA) publishing requirements from funders given the high costs of Article Processing Charges (APCs). As part of a broader effort to investigate effective and sustainable avenues of support for APCs, the library’s Scholarly Communications Committee created a novel, interactive dashboard (https://www2.hshsl.umaryland.edu/hshsl/about/openaccess.cfm) to track the university’s OA publications and gain a better understanding of OA trends and barriers. The dashboard was embedded into the library website, making it accessible to the entire university community. The goal of the dashboard project was to create an accessible, sustainable product that could be shared with and customized for multiple campus stakeholders.

## DESCRIPTION

To achieve the project goals, publication data were exported from Scopus and imported to Microsoft's Power BI to build the dashboard, where facets including OA model, publisher, journal, year, and school can be used to create different visualizations. To keep the dataset manageable, the project team focused on publications from the last five years.

The dashboard was designed iteratively and collaboratively, from planning starting in Summer 2024, to collecting and modeling the data in Fall 2024, and finally refining and publishing the visualization in Spring 2025. Since the Scholarly Communications committee membership changes annually, the 2024 committee prioritized automating the process of updating the dashboard with new data where possible so that future committees can continue to refine the dashboard based on feedback from stakeholders.

**Figure 1:**
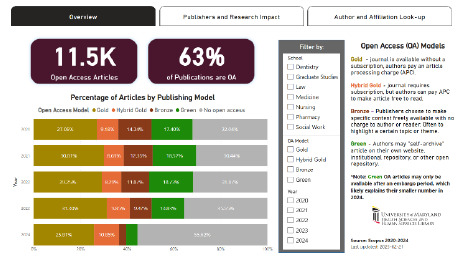
Overview

**Figure 2:**
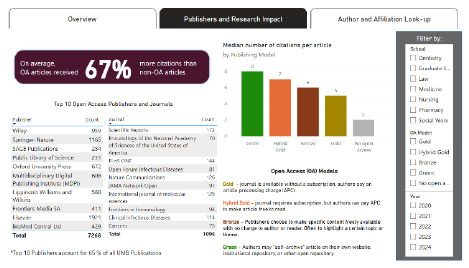
Publishers and Research Impact

**Figure 3:**
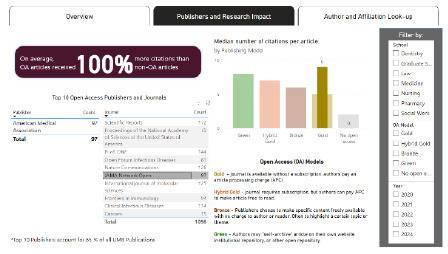
Publishers Filtered

**Figure 4:**
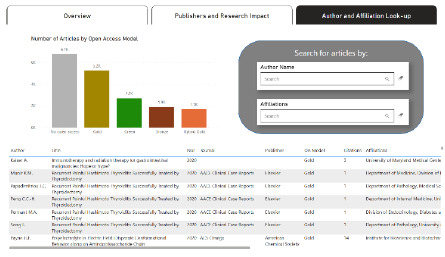
Author Lookup

## TECHNOLOGY

The dashboard was built in Power BI (Version 2.141.1253.0), a Microsoft data visualization and business intelligence platform. Power BI allows data to be imported in multiple formats. This data can be modeled and cleaned within the platform using the embedded Power Query technology. The dashboard was designed in Power BI Desktop and then published to the cloud-based Power BI service. From there, it was embedded into the library website. By using Power BI, the project team was able to take advantage of an institutional Microsoft account and connect the dashboard to the data stored in an Excel file on a shared Microsoft Teams site.

## CONCLUSIONS

The dashboard provides valuable insights: nearly two-thirds of UMB's publications from the past five years (that are indexed in Scopus) are OA, and those OA publications received more citations on average than non-OA publications (a median of 8 citations for Green OA versus 2 citations for non-OA). The dashboard approach has the advantage of allowing stakeholders to customize the visualizations based on their interests. The project team created a documented workflow (https://osf.io/qcw2p/), ensuring that future library committees will easily be able to update data and implement future changes. However, limitations remain: the data are constrained to publications indexed in Scopus, a database selected because it provides both affiliation and open-access information. Additional integration with surveys, APC data, and other sources is also needed to present a fuller picture of publishing practices, which is something the project team hopes to incorporate into the dashboard in the future. The project team also plans to investigate ways to further automate the process of updating the dashboard's data using the Scopus API.

The dashboard has received positive feedback in presentations to library leadership and campus organizations such as the Institute for Clinical and Translational Research. While current usage data is low, 142 page views since April 2025, the library communications team is seeking additional opportunities to promote the dashboard to a wider campus audience. By offering a replicable, well-documented process for visualizing OA publishing trends, the project supports informed decision-making within the institution while also serving as a model for other health sciences libraries seeking to advance their own OA initiatives.

**Contact Information: publishing@hshsl.umaryland.edu**

## DOCUMENTATION

https://osf.io/qcw2p/
